# Microwave Dual-Crack Sensor with a High Q-Factor Using the TE_20_ Resonance of a Complementary Split-Ring Resonator on a Substrate-Integrated Waveguide

**DOI:** 10.3390/mi14030578

**Published:** 2023-02-28

**Authors:** Yelim Kim, Eiyong Park, Ahmed Salim, Junghyeon Kim, Sungjoon Lim

**Affiliations:** 1 School of Electrical and Electronics Engineering, Chung-Ang University, Seoul 06974, Republic of Korea; 2 School of Engineering, University of British Columbia, Kelowna, BC V1V 1V7, Canada

**Keywords:** complementary split ring resonator, metamaterial-based sensor, quality factor, multi-crack detection, higher-mode substrate-integrated waveguide

## Abstract

Microwave sensors have attracted interest as non-destructive metal crack detection (MCD) devices due to their low cost, simple fabrication, potential miniaturization, noncontact nature, and capability for remote detection. However, the development of multi-crack sensors of a suitable size and quality factor (Q-factor) remains a challenge. In the present study, we propose a multi-MCD sensor that combines a higher-mode substrate-integrated waveguide (SIW) and complementary split-ring resonators (CSRRs). In order to increase the Q-factor, the device is miniaturized; the MCD is facilitated; and two independent CSRRs are loaded onto the SIW, where the electromagnetic field is concentrated. The concentrated electromagnetic field of the SIW improves the Q-factor of the CSRRs, and each CSRR creates its own resonance and produces a miniaturizing effect by activating the sensor below the cut-off frequency of the SIW. The proposed multi-MCD sensor is numerically and experimentally demonstrated for cracks with different widths and depths. The fabricated sensor with a TE_20_-mode SIW and CSRRs is able to efficiently detect two sub-millimeter metal cracks simultaneously with a high Q-factor of 281.

## 1. Introduction

Surface cracks in the metal caused by fatigue and corrosion, which can lead to significant problems in both small metallic objects and large structures, are difficult to detect and repair. Recently, various metal crack detection (MCD) techniques have been developed, including destructive tests such as acid corrosion, sulfur printing, and pinhole tests. However, non-destructive testing (NDT) has received significant attention because destructive tests can cause undesired damage and are limited in detecting cracks under coatings, such as rust, paint, corrosion protection, and composite laminate [1,2,3]. NDT techniques include advanced ultrasound, acoustic emissions, eddy currents, and optical fiber techniques, all of which offer high-resolution crack detection. However, they have disadvantages such as high complexity, high cost, large-sized equipment, and manufacturing difficulties [4,5,6,7,8,9,10,11,12]. In contrast, microwave-based NDT employing microstrips, waveguides, meta-resonators, radio-frequency (RF) identification, and antennas are low-cost, have a smaller sensor size, and allow for easier fabrication [13,14,15,16,17,18,19,20]. Despite these advantages, microwave-based NDT is limited in its ability to detect a variety of crack shapes and multiple cracks simultaneously and generally requires an increase in sensitivity.

The quality factor (Q-factor) is a key parameter for sensitivity; thus, various RF sensor studies have sought to develop sensor systems with a high Q-factor [21,22,23]. High Q-factor RF sensors can be realized using active or passive feedback systems. Assisting an active feedback loop using an active microwave device can dynamically increase the Q-factor; however, DC adjustment and calibration are important issues for this setup that need to be solved. Another problem associated with active microwave sensors is their limited operating temperature range [24,25,26,27,28,29]. On the other hand, passive structures with high Q-factor are generally implemented using reduced capacitance or increased inductance, complicating the structure. As a result, it is difficult to simultaneously detect multiple cracks and produce a high Q-factor because multi-crack detection sensors that requires independently operating resonances have unique structures such as multiband filters or multiband antennas [30,31,32,33,34]. Sensors that can detect two materials simultaneously and independently have gained significant research attention because they can significantly increase the sensing accuracy and reduce the scanning time for large areas while reducing power consumption [35,36,37,38,39,40,41,42,43,44,45,46]. In this paper, we propose a novel method that can increase the number of cracks that can be detected and produce a high Q-factor by integrating complementary split-ring resonators (CSRRs) with a higher-mode substrate-integrated waveguide (SIW).

A CSRR is a metamaterial structure that has an etched SRR on the opposite side of the signal line. Metamaterials (MM) are materials with the negative property of permeability and were first shown experimentally in 1999 as split ring resonators (SRR). The SRR and CSRR have been used for various RF applications such as antennas, sensors, filters, and transmission lines [46,47,48]. In CSRR, we can control the inductance and capacitance by the slots and line of CSRR. For example, when a voltage difference is excited in the CSRR, capacitance is induced in the slot by the electric field (E-field), and inductance is induced in the metal strip and conducting island by the magnetic field (H-field). For example, when a voltage difference is excited in the CSRR, capacitance is induced in the slot by the electric field (E-field), and inductance is induced in the metal strip and conducting island by the magnetic field (H-field) [49,50,51]. CSRRs can be used for multiple sensing because, when independently patterned CSRRs are employed, each pattern generates its own resonance frequency. In [42], sixteen CSRRs were loaded on four signal lines for multi-sensing, and in [45], coupled CSRRs were used for a dual- and triple-notch filter, and three CSRRs with a cavity were used in [52]. Additionally, the external field and Q-factor (~100) of CSRRs are helpful for MCD. Despite the already high Q-factor for CSRRs, various studies proposed new CSRR structures to increase the frequency-shifting sensitivity or the Q-factor further [52,53,54,55,56,57,58,59,60,61,62,63]. In [56,59,64], increasing the capacitance or inductance of the CSRR was used to increase the frequency-tuning sensitivity, and a higher Q-factor was realized by increasing the number of CSRRs [54,62,63] and using a T-shaped resonator [64,65,66,67].

In this paper, we produce a high Q-factor (~200) for a multi-crack sensor by integrating CSRRs with a higher-mode SIW. A SIW is a planar structure that offers additional functionalities over traditional waveguides, and attempts have been made to combine meta-material structures with a SIW to increase the Q-factor and sensitivity or reduce the sensor size [68,69,70,71,72,73,74]. However, combining CSRRs with a higher-mode SIW to separate and concentrate E-fields is proposed for the first time in the present study to enable multi-crack detection with a high Q-factor. When the independent CSRRs are loaded onto the SIW, each CSRR develops a stronger E-field distribution exterior to the SIW plane at each resonance frequency. To demonstrate our approach, we fabricated a dual-crack sensor using two CSRRs on a TE_20_-mode SIW. We loaded one CSRR at each hotspot to enhance the individual E-fields and simultaneously detected two metal cracks. The observed results for the proposed crack sensor confirmed that the sensor could detect different crack widths and depths simultaneously and independently.

## 2. Proposed Sensor Design

**Figure 1:**
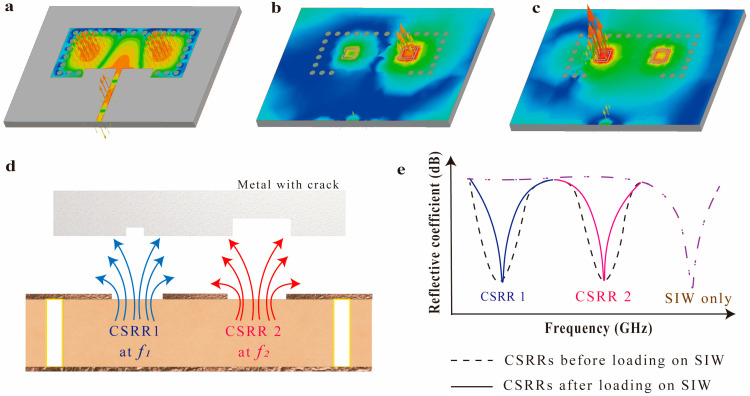
Proposed multi-crack detecting sensor with a high Q-factor: (**a**) TE_20_ mode SIW only E-field vector distribution. E-field vector distribution after the integration of the CSRRs (**b**) at 4.69 GHz and (**c**) 5.49 GHz. The E-field intensity range of (**a**–**c**) is the same. Concept illustration of (**d**) independent multi-crack detection and (**e**) the reflective coefficients before and after CSRR loading.

Figure 1 summarizes the principles underlying the proposed multi-crack detection sensor with a high Q-factor. First, we used a higher-mode SIW to form a separated E-field distribution, and the SIW subsequently enhanced the Q-factor of the CSRRs (Figure 1a). Next, independent CSRRs were positioned in the separated and concentrated E-fields on the TE_20_-mode SIW. Each CSRR produces an independent resonance, and the resonance frequency strengthens the E-field of the SIW (Figure 1b,c). As a result, changes in the metal surface, such as cracks or slots, sensitively affect the reflective coefficient due to the stronger external field for each CSRR (Figure 1d).

For example, when a crack is near CSRR1, the field near this CSRR changes, affecting the reflective coefficient for its resonance frequency. On the other hand, when a crack is near CSRR2, the crack only affects the reflective coefficient for the resonance frequency of CSRR2. When cracks are near both CSRRs, the reflective coefficients both change. In particular, loading the CSRR onto a higher-mode SIW has some advantages. Firstly, a higher-mode SIW enhances the Q-factor of the CSRR by strengthening the electromagnetic field; thus, the proposed sensor has a sub-millimeter sensing resolution and can distinguish between various crack shapes. Secondly, the use of CSRRs reduces the physical size of the SIW while maintaining its advantages because the higher capacitance and inductance of the CSRR decrease the operating frequency of the SIW (Figure 1e).

Table 1 compares the performance of the proposed crack sensor with that of conventional multi-detection RF sensors and crack detection sensors using CSRRs. These approaches have received significant attention because multi-detection capability significantly reduces the scanning time over wide areas and reduces power consumption. In [44], a power divider was used to produce independent frequencies and increase the Q-factor, leading to the fabrication of a sensor with high accuracy for liquid material permittivity measurements. In [16], a substrate highly sensitive to temperature was used to combine temperature and crack sensing. In [18], metal crack and strain sensing were achieved using a dual-mode passive and active antenna.

For MCD, many researchers have focused on increasing the Q-factor or the number of cracks that can be detected, for which antennas and CSRRs are widely used. We previously demonstrated an increase in the Q-factor by integrating a CSRR with the dominant mode of a SIW [35]. In [36], four SRRs were used to increase the detectable number of cracks, exhibiting high sensitivity at 38 GHz. However, SRRs create E-fields on the surface, and some exhibit unstable detection performance with a sensing resolution that varies from 0.1 mm to 0.5 mm, depending on the crack position. In [14,41], higher-mode antennas were introduced to produce independent frequencies and detect the tilting angle of metal cracks. In [44], a spoof surface plasmon polariton sensor was used for multiple crack detection by incorporating liquid channels. The sensor had high accuracy, sensing resolution, and sensitivity, but liquid switches have a slow switching speed and require an additional system for movement and storage. Although the sensor in [13] had the capability to detect 12 cracks simultaneously and independently, it had a low Q-factor and resolution. In contrast, the highly sensitive sensor proposed in the present study has a high Q-factor and sensing resolution in terms of both width and depth. In particular, the sensor can detect crack depths at a resolution of 0.2 mm due to the stronger external E-field.

### 2.1. Design of the Proposed Dual-Crack Detection Sensor

**Figure 2:**
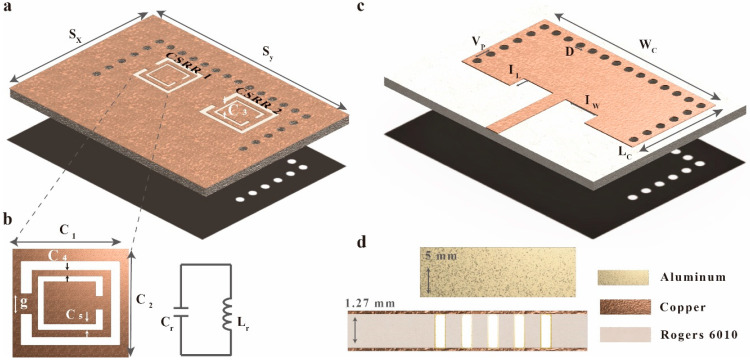
Proposed dual-crack detection sensor: (**a**) top view, (**b**) CSRR structure and equivalent circuit, (**c**) bottom view, and (**d**) side view.

Figure 2 presents the proposed dual-crack sensor design. It consists of a three-layered bottom conductive element with a signal line, a top conductive layer with two etched CSRRs on the ground plane, and a substrate with a via that connects the top and bottom plane to produce SIW cavities. We used a 1.27 mm thick Rogers Duroid 6010 substrate with a dielectric constant ($\varepsilon_{r})$ of 10.2 and a loss tangent (tan δ) of 0.0023. Additionally, the final sensor design included an uncracked metal sheet (Figure 1c). In this paper, aluminum with a thickness of 5 mm was used by a metal sheet. 

The geometrical parameters for the SIW and CSRRs were determined using Equations (1)–(8). First, we designed the TE_20_-mode SIW structure using Equations (1)–(3). The resonance frequency of the TE_20_-mode SIW was calculated as

(1) $$f_{mn0}=\frac{1}{2{\sqrt{\mu \varepsilon }}^{\;}}\sqrt{{\left(\frac{m}{W_{eff}}\right)}^{2}+{\left(\frac{n}{L_{eff}}\right)}^{2}},$$

where$\;\varepsilon ,\;\;\mu ,\;\;m,\;\;n,\;\;W_{eff},\;$and $L_{eff}\;$ are the substrate permittivity, permeability, mode m index, mode n index, and effective width and length, respectively. The effective width and length were calculated as

(2) $$W_{eff}=W_{c}-1.08\frac{D^{2}}{V_{p}}+0.1\frac{D^{2}}{W_{C}}\;$$

(3) $$L_{eff}=L_{c}-1.08\frac{D^{2}}{V_{p}}+0.1\frac{D^{2}}{L_{c}}\;$$

where $W_{c}$ and $L_{c}$ are the width and length of the SIW, respectively, from the center of the vias, and D is the diameter of the vias [47,74]. Based on the SIW, the TE_20_-mode resonant frequency for the SIW loaded with CSRRs can be expressed as

(4) $$f_{\mathrm{CSRR}}=\frac{1}{2\pi {\sqrt{L_{r}C_{r}}}^{\;}}\;$$

where $L_{r}$ and $C_{r}$ are the inductance and capacitance of the CSRR as shown in Figure 2b [74,75], which are determined by Equations (5) and (6), respectively:

(5) $$L_{r}=\frac{4.86\mu_{0}}{2}\left(c_{1}-c_{4}-c_{5}\right)\left[\ln\left(\frac{0.98}{\rho }+1.84\rho \right)\right]\;$$

(6) $$C_{r}=\left(c_{1}-1.5\left(c_{4}+c_{5}\right)\right)C_{pul}\;$$

where $\rho =\frac{c_{4}+c_{5}}{c_{1}-c_{4}-c_{5}}$ and $C_{pul}$ is the capacitance per unit length between the rings [76,77,78,79,80].

In addition, the proposed sensor employs CSRRs to reduce the evanescent wave effects and obtain a higher Q-factor compared with SIW-only approaches. The Q-factor at each resonant frequency can be expressed as

(7) $$Q=R\sqrt{\frac{C_{r}}{L_{r}}}\;$$

where $C_{r}$ and $L_{r}$ are defined in Equations (5) and (6), respectively. A higher Q-factor increases the sensitivity, i.e., sharper resonance E-field concentrations and perturbations [77]. A large D and $V_{p}$ can occur due to E-field leakage in the SIW, thus we limit these to reduce radiation losses as follows:

(8) $$D<\frac{\lambda_{g}}{5}\;,\;V_{p}\leq 2D,\;$$

where $\lambda_{g}$ is the guided wavelength.

Finally, the SIW cavities within the substrate have a width W of 25 mm, a length L of 11.1 mm, a via diameter D of 1 mm, and a center-to-center separation $V_{p}$ of 1.8 mm (Figure 1d). The two CSRRs etched on the bottom of the sensor have a gap g of 0.5 mm and an external and internal ring width and ring spacing ($c_{3}$, $c_{4}$, and $c_{5}$, respectively) of 0.2 mm each. The external square width and length ($c_{1}$ and $c_{2}$) are both 2.7 mm for CSRR1 (left side, Figure 2a) and both 3.1 mm for CSRR2 (right side, Figure 2a), with the other parameters the same for the two CSRRs. The cavities are connected to a 50-Ω microstrip line with a width of 1 mm and a length of 10 mm that links to the subminiature version A (SMA) connector. The cavity inserts were designed to match the impedance with the aluminum sheet used as the CSRR loading plane, with a width $I_{W}$ of 6 mm and a length $I_{L}$ of 2 mm. The total substrate size is SX = 36 mm and SY = 26 mm.

### 2.2. Sensing Method

**Figure 3:**
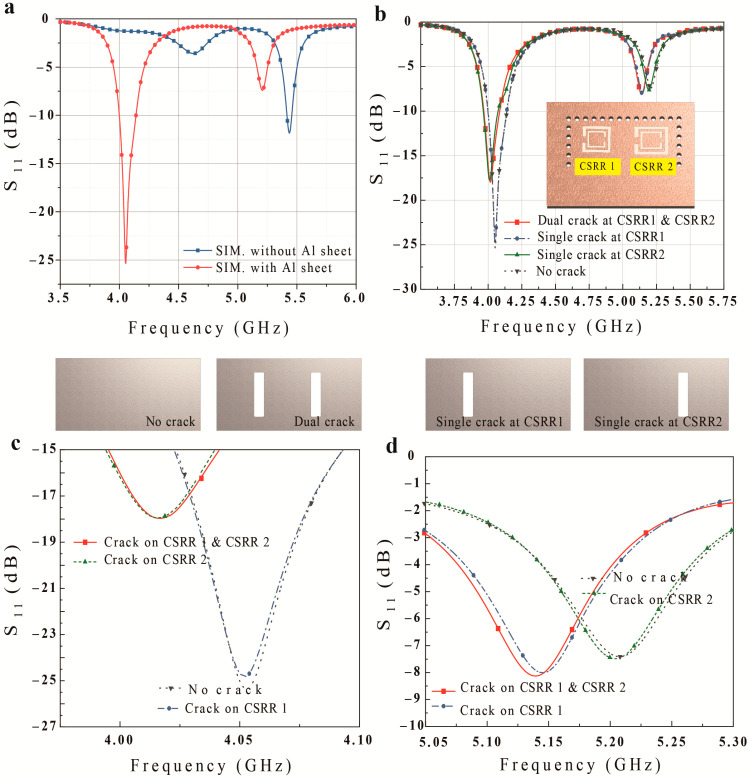
Simulation results for the proposed crack sensor: (**a**) S11 without an Al sheet and with an uncracked Al sheet, (**b**) S11 for different crack numbers and positions (two cracks, single crack at CSRR1, single crack at CSRR2, no crack), S11 results (**c**) in the CSRR2 resonance frequency band, and (**d**) in the CSRR1 resonance frequency band (crack width, depth, and length of 0.5 × 0.5 × 6.1 mm).

Figure 3 presents simulated S11 results for the proposed crack sensor. The resonant frequency of the sensor changes from 4.626 and 5.436 GHz to 4.053 and 5.208 GHz after loading the Al sheet because the addition of material that has a different dielectric constant affects the resonant frequency (Figure 3a). In this paper, we optimized the sensor via the loading material to increase its sensitivity for surface cracks. Figure 3b−d show that the proposed sensor can detect the position of multiple cracks with a width, depth, and length of 0.5 × 0.5 × 6.1 mm^3^. For example, when cracks are above both CSRRs, the resonance frequencies change from 4.053 and 5.208 GHz to 4.020 and 5.145 GHz for CSRR1 and CSRR2, respectively. On the other hand, when a crack is only above CSRR1, only the resonance frequency for CSRR1 changes, while the same is true for CSRR2. This is because the E-field near the CSRR is disturbed by the material, affecting the resonant frequency of the CSRR. 

In addition, when a crack is present in the CSRR region, the capacitance is affected. The concentrated E-field near the CSRR flows along the crack, which operates as a capacitor. The shorter side of the crack is considered the crack width, and it can be considered the distance between capacitors. The crack depth can be considered the area of the capacitor. When the crack width increases, the capacitance decreases, and the resonant frequency increases. In addition, as the crack depth increases, the capacitance rises, and the resonant frequency falls. The resonant frequency of a CSRR in the presence of a crack is represented by Equation (9):

(9) $$f_{CSRR}=\frac{1}{2\pi {\sqrt{L_{r}(C_{r}+(C_{crack})}}^{\;}},\;$$

where $C_{crack}$ is the capacitance included by the crack. Thus, the proposed sensor detects a crack using the change in the resonant frequency. The proposed multi-crack sensor has a low fabrication cost and small waveguide cavities, and the independent sensing area can be extended because multi-E-fields in TEmn mode do not affect other sensing results [81,82,83].

**Figure 4:**
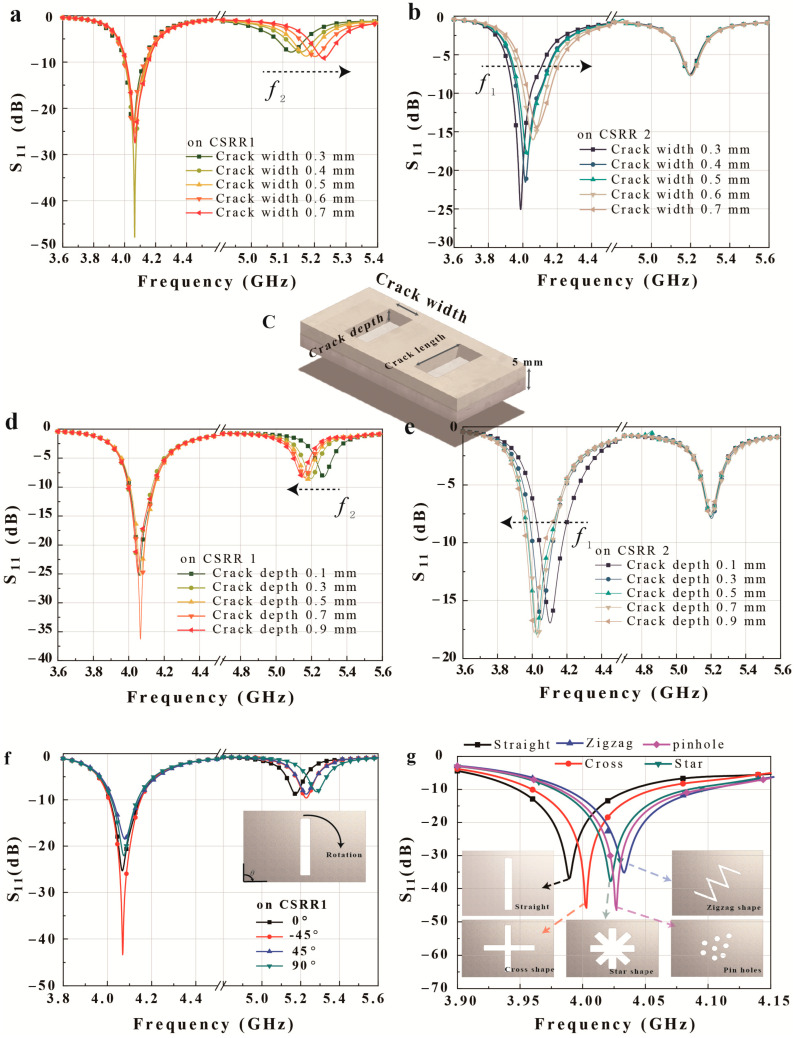
Simulated S11 results for the proposed dual-crack sensor for different crack widths above (**a**) CSRR1 and (**b**) CSRR2. (**c**) Fundamental metal sheet structure with two cracks with a width, depth, and length of 0.5 × 0.9 × 6.1 mm. S11 results for different crack depths above (**d**) CSRR1 and (**e**) CSRR2. (**f**) S11 results for the rotation of a straight crack above CSRR1. (**g**) S11 results for five crack shapes above CSRR2.

**Figure 5:**
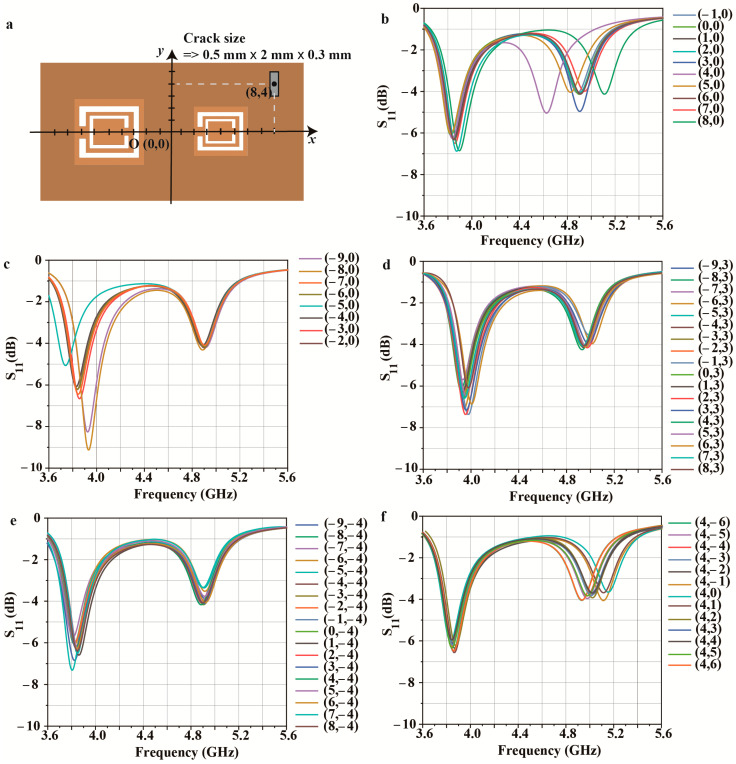
Simulation results for different crack locations (*x*, *y*) on the Al sheet: (**a**) coordinate of the crack; (**b**–**e**) S11 when the crack moves *x*-axis; (**b**) from (−9, 0) to (-2, 0), (**c**) from (−1, 0) to (8, 0), (**d**) from (−9, 3) to (8, 3), and (**e**) from (−9, 4) to (8, 4); (**f**) S11 when the crack move *y*-axis from (4, −6) to (4, 6).

**Figure 6:**
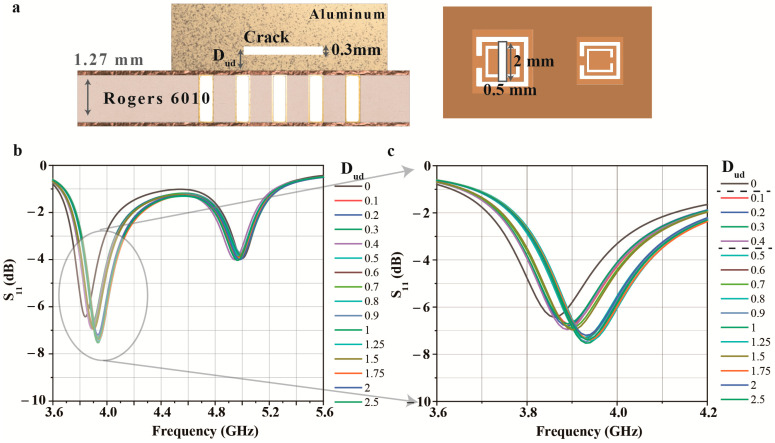
Simulation result of the proposed sensor for different depths from a metal surface: (**a**) side view and top view of structure and crack; (**b**,**c**) the simulated S_11_ for different crack positions: depth from a metal surface D_ud_ = 0–2.5 mm.

**Figure 7:**
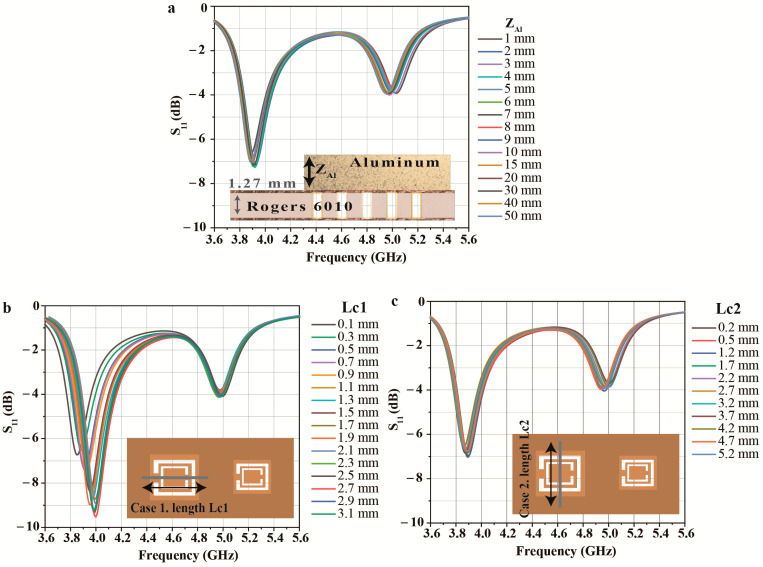
Simulation results of the proposed sensor for (**a**) different Al sheet thicknesses and (**b**,**c**) crack lengths.

Figure 4, Figure 5, Figure 6 and Figure 7 are numerical analyses to check the sensing capability. Figure 4a–e show the sensing resolution for different crack width when the crack is positioned in the center of each CSRR. The proposed sensor offers multi-crack sensing at a high sensing resolution based on the stronger Q-factor. As a result, the sensor has a detection resolution of 0.1 mm for width and 0.2 mm for depth in both positions.

Figure 4a,b display the simulated frequency responses for various straight crack widths, with a consistent length of 6.1 mm for all cracks. Figure 4a presents the simulated S11 results as the width of a single straight crack above CSRR1 increases from 0.3 to 0.7 mm at intervals of 0.1 mm with a fixed depth of 0.5 mm. The resonant frequency for CSRR1 increases from 5.126 to 5.229 GHz as the crack width increases, and the average change in frequency is 257.5 MHz/mm (Figure 4a). On the other hand, when the width of a single straight crack above CSRR2 increases from 0.3 to 0.7 mm at intervals of 0.1 mm with a fixed depth of 0.5 mm, the resonant frequency for CSRR2 increases from 3.988 to 4.083 GHz and the average frequency shift is 237.5 MHz/mm. Figure 4d,e present the simulated S11 results when the depth of a single straight crack above CSRR1 and CSRR2 increases from 0.1 to 0.9 mm at intervals of 0.2 mm. The resonant frequency of CSRR1 decreases from 5.269 to 5.145 GHz, and the average change in frequency is 142.5 MHz/mm when the crack depth is changed above CSRR1. In contrast, when the crack depths above CSRR2 increase, the resonant frequency of CSRR2 decreases from 4.103 to 4.011 GHz, and the average change in frequency is 132.5 MHz/mm. The simulation results thus demonstrate the sensitivity of the proposed sensor for the detection of straight cracks, with the ability to distinguish differences of 0.1 and 0.2 mm in width and depth, respectively.

The proposed dual sensor can detect various shapes at each CSRR due to its high sensitivity. Figure 4f,g present the simulated S11 results for five crack shapes commonly found on metal surfaces: tilted, cross, zigzag, star, and pinhole. Figure 4f presents the S11 results for different tilting angles above CSRR1. A ±45° rotation of the crack increases the resonant frequency to 5.228 GHz compared with a resonant frequency of 5.174 GHz at 0°. Similarly, a 90° horizontally oriented straight crack has a resonant frequency of 5.283 GHz. Figure 4g displays the S11 results for the straight, cross, zigzag, star, and pinhole cracks above CSRR2. Because the difference in the crack distribution affects Ccrack in Equation (9), and small differences can have a significant effect due to the concentrated E-field, the different crack patterns lead to differences in the change in the resonant frequency.

Figure 5 is the simulated result for checking the effect of the crack location deviation. In conclusion, since CSRR forms an electric field around the center of the ring, the frequency range varies depending on the location of the crack. First, Figure 5c,d show the simulated S11 of the proposed sensor for the *x*-direction position deviation of the crack. The original point (0, 0) is the intermediate position from the side of each CSRR. As shown in Figure 5b, when the crack moves from (−1, 0) to (8, 0) around CSRR2, the further away from (4, 0), the center of the CSRR, the smaller the range of frequency variation. In the same way, in Figure 5c, when the crack moves from (−9, 0) to (−2, 0) around CSRR1, the further away from (−5, 0), the center of the CSRR, the smaller the range of frequency variation. However, when the crack positions outside the CSRR where the electric field is weak, there is little change in frequency from the *x*-axis movement of the crack, as shown in Figure 5d,e. In addition, we checked the effect of the *y*-direction position deviation of the crack (Figure 5f). Similar to the change in the *x*-axis, it was shown that the closer the center of the CSRR, the greater the frequency change in the position deviation of the *y*-axis. For example, when the crack is in (4, 0), the frequency moves from 4.82 GHz to 5.18 GHz; when the crack is in (4, −1) or (4, 1), the frequency moves from 4.82 GHz to 5.1 GHz; and when the crack is in (4, −3) or (4, 3), the frequencies move from 4.82 GHz to 5 GHz. However, when the crack is outside the CSRR, there is little change in frequency from the *y*-axis movement of the crack.

Next, Figure 6 shows the detection capability of the crack under the metal surface. Generally, non-destructive testing can be used for detecting cracks under the surfaces. In the simulation, the crack of 0.5 × 2 × 0.3 mm^3^ is used, and the crack is positioned in the center of CSRR1, as shown in Figure 6a. Then, the crack is moved from the metal surface (D_ud_ = 0 mm) to the interior of the metal (D_ud_ = 2.5 mm). As a result, when D_ud_ changes from 0.1 mm to 0.4 mm, the frequency is changed from 3.82 GHz to 3.88 GHz, and when D_ud_ is bigger than 0.4 mm, the resonance frequency of the sensor generates at 3.94 GHz. In summary, the proposed sensor can detect whether a crack is on a metal surface or inside the metal, but it is difficult to distinguish how deep the crack penetrates accurately. 

Finally, Figure 7 shows the effect of the thickness of the Al sheet for the proposed sensor and the detection capability for the crack length. First, the thickness of the Al sheet has little effect on the RF sensor because the metal material reflects almost all the waves on the surface, as shown in Figure 7a. Next, the length of the crack has a different effect on the RF sensor depending on the direction of the crack. Figure 7b shows that a change in frequency occurs when the crack is lengthened toward the gap of the ring since the electric field is strongly formed in the center of the CSRR and near the gap of the ring. Conversely, little frequency change appears when the crack is lengthened in a direction unrelated to the ring’s gap, as shown in Figure 7c.

## 3. Fabrication of a Prototype Sensor and Measurement

### 3.1. Fabrication and Sensor Measurement Results

**Figure 8:**
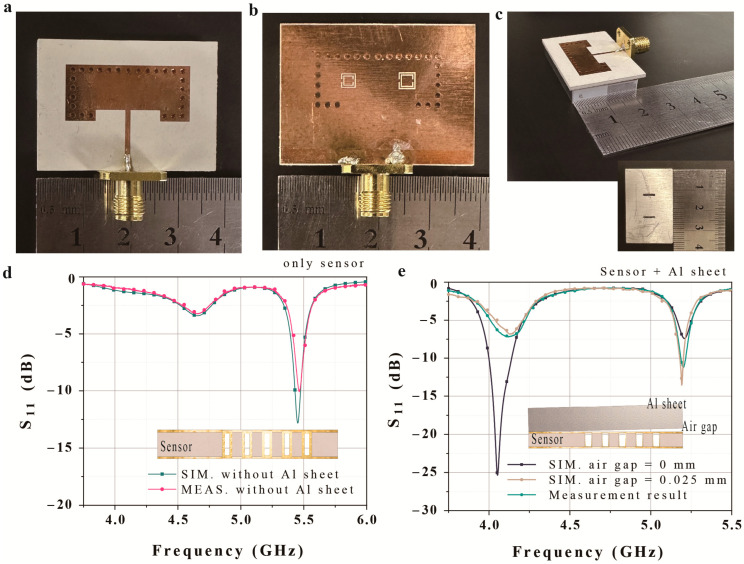
Fabricated prototype of the proposed dual-crack sensor: (**a**) top view, (**b**) bottom view, and (**c**) side view with the Al sheet. Simulation and measurement results for (**d**) the sensor with no Al sheet and (**e**) the sensor with an uncracked Al sheet.

We fabricated a prototype SIW-based dual-crack sensor with various Al sheets in accordance with the proposed design. Figure 8a,b show top and bottom views of the fabricated sensor, and Figure 8c shows a side view of the fabricated sensor with the Al sheet. We employed four representative Al sheets with different straight crack widths and depths (Table 2); more complex shapes were not tested due to the difficulty of fabrication.

The Al sheets were the same size as the detector (36 mm × 23 mm width and length) and were 5 mm thick. Because the cracks were positioned symmetrically, we measured eight situations using the four samples. The width and depth were changed, while the distance between the cracks was 10.8 mm, and the crack length was 6.1 mm. The fabricated cracks are located at the center of the CSRRs.

Figure 8c,d compares the simulation and measured sensor results for the sensor only and for the sensor with an uncracked aluminum sheet. The S-parameter of the fabricated sample is measured using a Keysight N5227B (Keysight, Santa Rosa, CA, USA) vector network analyzer. Figure 8c shows that the measurement results for the sensor without an Al sheet are consistent with the simulation results. However, although the sensor with an uncracked Al sheet exhibits a similar trend, there is some inconsistency with the simulation results, especially in terms of the magnitude of S11. This is ascribed to the presence of a small air gap between the prototype sensor and the Al sheet (Figure 8e). When we employed a 0.025 mm air gap between the sensor and Al sheet in the simulation, the results became consistent.

### 3.2. Crack Sensing Performance

**Figure 9:**
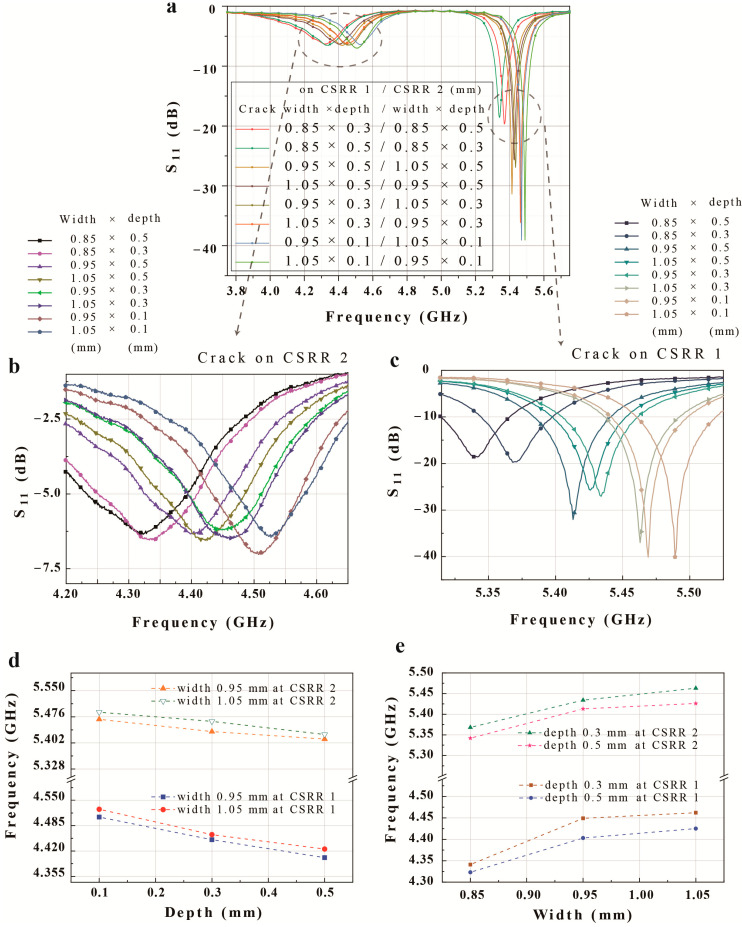
Measured S-parameters for the proposed dual-crack sensor (**a**) with various slot widths and depths at (**b**) CSRR 2 and (**c**) CSRR 1. Measured relationship between the frequency and various crack sizes: (**d**) depth and (**e**) width.

Figure 9a presents the change in the resonant frequency due to the eight types of crack for each CSRR. In the absence of any cracks, the resonance frequency of the sensor is 5.20 GHz for CSRR1 and 4.12 GHz for CSRR2. In the presence of a crack with a width of 0.85 mm and a depth of 0.5 mm, the frequency increases to 4.32 GHz for CSRR2 and 5.34 GHz for CSRR1. In addition, as the width of the crack increases, the resonance frequency increases. On the other hand, when a crack has the same width, the resonance frequency increases as the depth of the crack reduces. Figure 9b,c present a more detailed plot of the measurement results for each CSRR, and the resonant frequency for each crack is summarized in Table 3.

Figure 9b displays the measured S11 plotted against the CSRR2 resonant frequency for the different cracks. The resonant frequency ranges from 4.320 GHz for the 0.85 × 0.5 mm crack to 4.527 GHz for the 1.05 × 0.1 mm ^2^ crack. This demonstrates that the proposed sensor can detect cracks above CSRR2 at a resolution of 0.1 mm for the crack width and 0.2 mm for the crack depth. Figure 9c presents the measured S11 plotted against the CSRR1 resonant frequency for the different cracks. The resonant frequency ranges from 5.340 GHz for the 0.85 × 0.5 mm^2^ crack to 5.489 GHz for the 1.05 × 0.1 mm^2^ crack; thus, CSRR1 has the same sending resolution as CSRR2.

Figure 9d,e show the relationship between the resonant frequency and crack width and depth. Figure 9d presents the effect of the depth on the sensing of a crack with a fixed width of 0.95 and 1.05 mm at each CSRR. The average change in the resonant frequency is 20.7 and 18.3 MHz for CSRR1 and CSRR2, respectively, with a change of 0.1 mm in width. The fabricated sensor exhibits a linear reduction in the resonant frequency at both CSRRs with an increasing crack depth. Figure 6e presents the effect of the width on crack detection with a fixed depth of 0.3 and 0.5 mm at each CSRR. The average change in the resonant frequency is 28.0 and 33.7 MHz for CSRR1 and CSRR2, respectively, with a change of 0.2 mm in depth. The fabricated sensor thus exhibits a linear increase in the resonant frequency with an increase in the crack width.

The proposed sensor experiences a decrease in the resonant frequency of 30.8 MHz when the crack width increases by 0.1 mm and an increase of 19.5 MHz when the crack depth increases by 0.2 mm. Thus, the proposed crack sensor can detect differences in crack width down to 0.1 mm and crack depth down to 0.2 mm.

The measured changes in the resonance due to changes in the crack dimension are generally linear, and the sensor can successfully simultaneously detect two different crack widths or depths, with each CSRR independent of the other. Cracks can thus be detected in situations where one crack is located near CSRR1, and another crack is located near CSRR2. In this respect, the measurement results exhibit the same trend as the simulation results and electromagnetic theory. The simulation results confirm that the effective capacitance falls with an increasing crack width; hence, the resonant frequency increases. In contrast, the effective capacitance increases with increasing crack depth, reducing the resonant frequency.

## 4. Conclusions

This study proposed a non-destructive multi-crack detection sensor consisting of differently sized CSRRs in a higher-mode SIW cavity to provide multi-resonant frequencies. Each CSRR on the SIW creates its own resonance, and the higher-mode SIW offers separated and concentrated E-fields for each CSRR. As a result, the proposed sensor has a high Q-factor and can simultaneously and independently detect multi-cracks. The reflective coefficient of the sensor is affected by the metal surface due to the strong external E-field from the CSRRs and SIW. When the metal has cracks, the efficient capacitance changes, which alters the resonant frequency of the nearby CSRR. Additionally, due to the high Q-factor, the proposed sensor can detect the metal crack at a high resolution. The effective capacitance of the sensor falls as the crack width increases, raising the resonant frequency, with the capacitance increasing as the crack depth becomes shallower, thus reducing the resonant frequency. The relationship between the resonance and the crack width or depth is almost linear. To demonstrate our proposed design, we designed and fabricated a dual-crack detection sensor using two independent CSRRs and a TE_20_-mode SIW. The fabricated crack sensor distinguishes crack width differences down to 0.1 mm and crack depth differences to 0.2 mm and is able to two detect two differently sized cracks independently and simultaneously with significantly lower power consumption and scanning time. The proposed dual-crack detection sensor also exhibits a higher Q-factor than previously reported multi-detection sensors.

## Figures and Tables

**Table 1:** Summary of the proposed and previously reported multi-crack detection sensors.

[Ref] Year	No. of Cracks Detected	Sensing Target	Sensing Techniques	Crack Sensing Resolution (mm)		Sensitivity (MHz/mm)		Measured Q-Factor
				Width	Depth	Width	Depth	
[39] 2020	2	Liquid material permittivity	Power divider + SRRs	N/A	N/A	N/A	N/A	280
[16] 2021	2	Temperature and metal cracks	Antenna + temperature-sensitive substrate	0.10	N/A	16.6	N/A	17
[18] 2020	2	Metal cracks and strain	Passive and active mode antenna	0.25	N/A	50	N/A	N/A
[35] 2012	1	Metal cracks	CSRR	0.10	N/A	200	N/A	25
[50] 2017	1	Metal cracks	SIW + CSRR	0.20	500	50	1	224*
[36] 2021	4	Metal cracks	Four SRRs	0.10−0.50	N/A	1300	N/A	N/A
[40] 2018	2	Metal cracks	Higher-mode patch antenna	1.00	N/A	140	N/A	N/A
[14] 2019	2	Metal cracks	Higher-mode patch antenna	0.20	N/A	45	N/A	N/A
[44] 2022	4	Metal cracks	Spoof surface plasmon polariton sensor + liquid switch	0.10	N/A	200	N/A	N/A
[42] 2021	12	Metal cracks	CSRRs	0.40	0.2	N/A (magnitude)		17
This work	2	Metal cracks	CSRRs + higher-mode SIW	0.10	0.2	200 *	130 *	281 *

* Highest value when the minimum crack is loaded.

**Table 2:** Crack sizes on the Al sheets.

Sheet	Crack Width (mm)	Crack Depth (mm)
1	0.85	0.3/0.5
2	0.95/1.05	0.1
3	0.95/1.05	0.3
4	0.95/1.05	0.5

**Table 3:** Proposed sensor performance for each CSRR.

Crack Width × Depth (mm)								
	0.85 × 0.5	0.85 × 0.3	0.95 × 0.5	1.05 × 0.5	0.95 × 0.3	1.05 × 0.3	0.95 × 0.1	1.05 × 0.1
CSRR1 *	5.34	5.368	5.413	5.426	5.434	5.463	5.469	5.489
CSRR2 *	4.32	4.341	4.403	4.425	4.449	4.462	4.507	4.527

* Unit is GHz.

## Data Availability

Not applicable.
